# Evaluation of Anatomical Measurements of the Bulbus Oculi by Optical Biometry in the Eastern Region of Türkiye

**DOI:** 10.3390/medicina61040692

**Published:** 2025-04-10

**Authors:** Methiye Batur, Veysi Yıldız, Muhammed Batur, Erbil Seven, Serek Tekin

**Affiliations:** 1 Department of Anatomy, Health Sciences Institute, Ataturk University, 25030 Erzurum, Turkey; 2 Department of Anatomy, Medical Faculty, Yuzuncu Yıl University, 65090 Van, Turkey; 3 Department of Ophthalmology, Medical Faculty, Yuzuncu Yıl University, 65090 Van, Turkey; 4 Urartu Eye Center, 65080 Van, Turkey

**Keywords:** eye anatomy, axial length, bulbus oculi, optical biometry

## Abstract

*Background and Objectives*: The objective was to assess ocular biometric measurements in relation to age and gender among patients scheduled for cataract surgery, utilizing an optical biometry device. *Materials and Methods*: The optical biometric parameters evaluated included axial length (AL), central corneal thickness (CCT), anterior chamber depth (AD), lens thickness (LT), horizontal corneal diameter (WTW), and keratometry values in the flat (K1) and steep (K2) meridians. Astigmatism (AST) was also measured as the difference between these keratometry values. *Results*: A total of 14,183 optical biometric measurements were included in the study. The average age of the participants was determined to be 56 (3–110) years. The average AL was 23.57 ± 1.45 mm, the average AD was 2.76 ± 0.42 mm, the average CCT was 518.13 ± 37.81 μm, the average WTW distance was 11.88 ± 0.59 mm, and the average LT was 4.19 ± 0.51 mm. The keratometry measurements were recorded as K1 = 43.39 ± 1.96 diopters (D), K2 = 44.51 ± 2.31 D, and AST = 1.12 ± 1.15 D. The average values for male eyes in terms of the AL, AD, WTW, and LT measurements were significantly higher than those for female eyes (*p* = 0.001). The average K1 and K2 values were flatter in males than in females, while the AST value was found to be higher in females (*p* = 0.001). As age progressed, the mean AL decreased, the CCT decreased, the AD narrowed, the WTW distance decreased, the LT increased, and the keratometric values K1, K2, and AST decreased until the age of 60, after which they increased again. *Conclusions*: Research indicates that the measurements of the bulbus oculi are generally larger in males than in females. Furthermore, each of the optical biometric measurements is interrelated. Over time, these measurements may change.

## 1. Introduction

Widely accepted knowledge exists in the medical literature regarding eye anatomy and the measurements of eye structures. However, recent advancements in the diagnosis and medical–surgical treatments of eye diseases have increased the need for more detailed and accurate data on eye measurements. Recent innovations in surgical techniques and intraocular lens designs have significantly improved the postoperative refractive outcomes of modern cataract surgery, increasing patients’ expectations for good vision without glasses. This outcome depends on the accurate prediction of the implanted intraocular lens (IOL) power, which primarily relies on preoperative biometric data [1]. Myopia, which is rapidly increasing and predicted to become an epidemic in the coming years, is closely related to the biometric measurements of the eye. Studies have examined the prevalence of myopia in populations across different continents, revealing a higher number of myopic eyes in Asian populations [2]. Studies on the relationship between diabetic retinopathy and ocular biometry have highlighted the importance of further biometric studies to elucidate the impact of high myopia and ocular biometry on the protective mechanisms of the eye [3].

Biometry involves the measurement of numerous parameters, the most important of which are axial length (AL), central corneal thickness (CCT), anterior chamber depth, lens thickness (LT), corneal diameter, and corneal curvature (keratometry). AL refers to the anterior–posterior diameter of the eye, measured at the center of the cornea. Keratometry represents the horizontal and vertical curvatures of the cornea. Variations in keratometry values across corneal meridians indicate the presence of corneal astigmatism.

A-scan ultrasound biometry, used to measure the axial length of the eye, was first introduced in 1956. This measurement is performed using an ultrasound probe placed at the center of the cornea. It measures the distance between the cornea and the inner limiting membrane of the retina. This method also provides anterior chamber depth and LT values, along with AL. However, a significant disadvantage of this method is the potential for anterior chamber shallowing due to inadvertent pressure on the cornea. This can lead to shorter eye measurements and higher estimations of intraocular lens power. Other disadvantages of A-scan ultrasound biometry include its low resolution, resulting in an AL measurement accuracy of approximately 100–200 μm. An error of 100 μm can result in a corresponding postoperative refractive error of 0.25 D. Inconsistent measurements can also occur due to retinal thickness inconsistencies in the central retina and off-axis measurements [4].

Optical biometry devices, introduced in 1999 (IOL Master, Carl Zeiss Meditec AG, Jena, Germany), are now widely available. One commonly used optical biometry device, the Lenstar LS900 (Haag-Streit AG, Koeniz, Switzerland), uses a non-contact measurement technique with an 820 nm wavelength laser diode infrared light. Optical biometry measures the distance from the cornea to the retinal pigment epithelium, which explains the inconsistencies in AL values obtained with ultrasound biometry. Optical biometry, with its higher resolution, offers several advantages over ultrasound biometry. Guidance for optical biometry is easier because the patient stabilizes the laser beam, whereas the orientation of the scan often requires an estimate for ultrasound measurements. Optical biometry simultaneously provides multiple parameters, including AL, CCT, anterior chamber depth, LT, keratometry values, corneal diameter, and astigmatism. Other advantages of optical biometry include its independence from the examiner, ease of application, and lack of infection risk. Currently considered the gold standard in ocular biometry, optical biometry has led to the development of more precise biometric systems [4].

Knowledge of the normal variations in ocular anatomy is essential for understanding the pathogenesis, diagnosis, and optimal management of ocular diseases. Ocular biometric parameters are known to vary with ethnicity. Therefore, studies have been conducted worldwide to define the ocular biometric characteristics of different populations [3,5,6]. According to our research, no large-sample studies have been published regarding biometric values for Türkiye. The aim of our study, which included a substantial sample size, was to evaluate ocular biometric measurements based on age and gender.

## 2. Material and Method

This retrospective study was conducted after obtaining approval from the Van Yüzüncü Yıl University Local Ethics Committee (Date: 20 September 2024, Number: 2024/10-09) and in accordance with the principles of the Declaration of Helsinki. Optical biometric measurements of both eyes of patients diagnosed with cataracts in one or both eyes between April 2016 and May 2024 were taken at the Van Yüzüncü Yıl University Department of Ophthalmology. Eyes that had undergone any eye surgery before, were pseudophakic or aphakic, had cataracts that were too advanced to be measured by optical biometry, or that had corneal pathology or vitreous–retinal pathology were not included in this study. Eyes for which the axial length or lens thickness could not be measured were also not included in this study.

**Figure 1:**
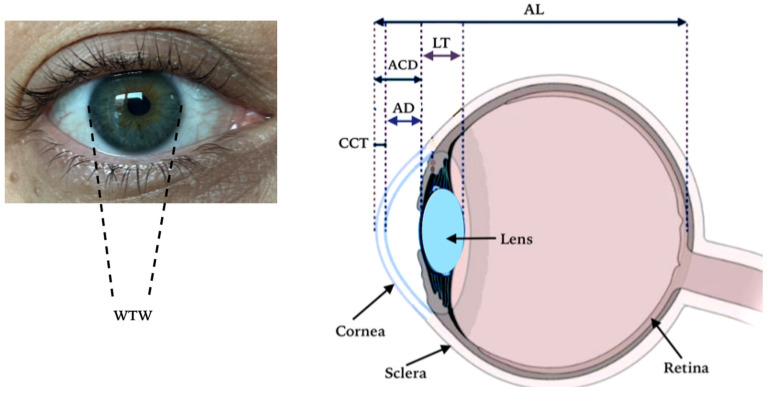
Optical biometry parameters. AL: axial length, CCT: central corneal thickness, AD: humor aqueous depth (between the corneal endothelium and anterior crystalline lens surface), WTW: white-to-white (horizontal corneal diameter), ACD: anterior camera depth (between the corneal epithelium and anterior crystalline lens surface), LT: lens thickness.

The patients’ age, gender, and optical biometric measurements of the right and left eyes were recorded separately. Among the optical biometry parameters (Figure 1), the AL, corneal center thickness (CCT), anterior chamber depth (humor aqueous depth; AD, distance between corneal endothelium and lens anterior capsule), LT, three to nine o’clock horizontal corneal diameter (WTW, white-to-white), anterior chamber depth (ACD, distance between corneal epithelium and lens anterior capsule), and flat (K1) and steep (K2) keratometry values in the meridian, along with the astigmatism (AST) value, which is the difference between these keratometry values, were recorded.

### 2.1. Optical Biometry Measurement (Lenstar LS900, Haag-Streit AG, Koeniz, Switzerland)

Patients who underwent a comprehensive ophthalmological examination, including visual acuity, anterior and posterior segment examination, and intraocular pressure measurement, and were diagnosed with cataracts in at least one eye, were included in optical biometry imaging. The optical biometric measurements were performed by experienced technicians in a dimly lit room. After ensuring that the patient focused on the fixation light of the biometry device, they were asked to close and open their eyes. An average of three separate optical biometric measurements was taken. Repeat measurements were performed for patients with low measurement quality.

### 2.2. Statistical Analysis

Descriptive statistics for the features emphasized are presented as the mean, standard deviation, standard error, confidence interval, and minimum and maximum values. In comparisons made between genders and between eyes (right eye OD, left eye OS) for these features, an independent group *t*-test was used. To determine the relationships among the features, correlation coefficients were calculated separately within the groups. The chi-square test was used to determine the relationships among the categorical variables. Statistical significance (significance level) was determined as 5% in the calculations and the SPSS (ver: 25) statistical package program was used for the calculations.

## 3. Results

**Figure 2:**
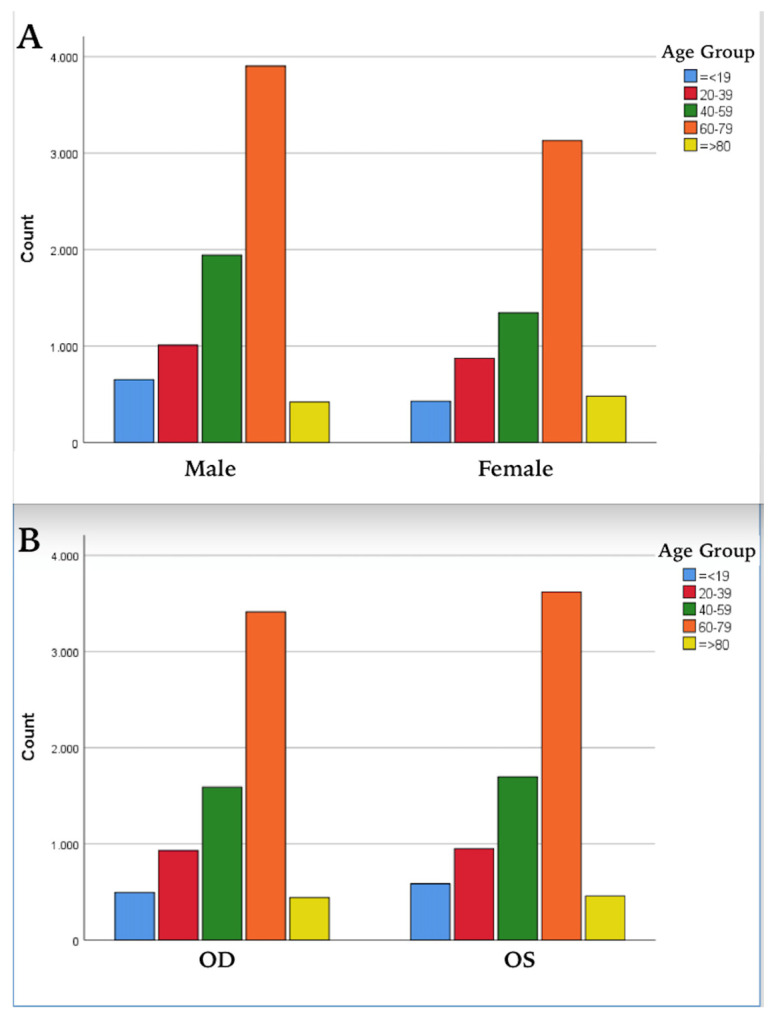
Distribution of subjects by age group, (**A**) gender (*p* = 0.001), and (**B**) right–left eye (*p* = 0.362). OD: right eye; OS: left eye.

The majority of the patients were from the eastern part of Türkiye, specifically Van Province and its five neighboring provinces: Ağrı, Bitlis, Hakkari, Muş, and Siirt. Optical biometric measurements were performed on 22,770 eyes on the specified dates. Considering the aforementioned exclusion criteria, a total of 14,183 eyes from 8409 patients were studied. Of these patients, 5774 (68.66%) were bilateral eyes, 1097 (13.05%) were right eyes only, and 1538 (18.29%) were left eyes only. The cases were grouped according to age ranges: 19 years and under, 20–39, 40–59, 60–79, and 80 years and older. The distribution of gender and right/left eye data across these age groups is presented in Table 1 and Figure 2.

**Figure 3:**
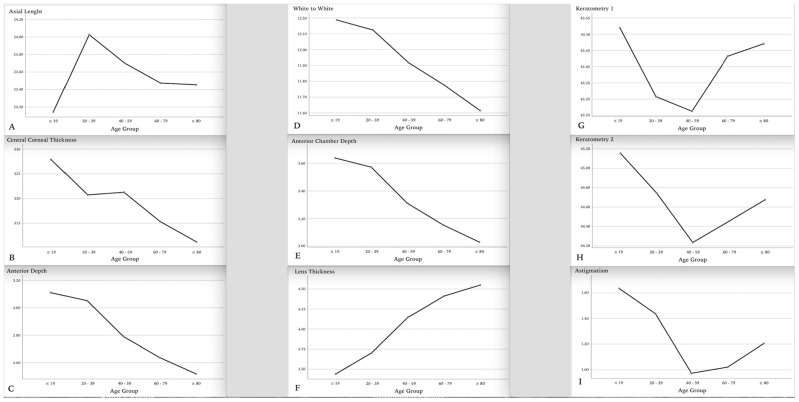
Optical biometric measurements by age group ((**A**–**I**), *p* = 0.001).

The optical biometry parameter measurements were found to vary across age groups. As age increased, the AL value decreased (except in the 19 years and under group), the CCT thinned, the AD narrowed, the WTW decreased, the LT thickened, and the K1, K2, and AST decreased toward age 60, and then increased again (Table 2, Figure 3).

Of the eyes included in the study, 7928 (55.9%) belonged to men and 6255 (44.1%) to women. The average values for the AL, AD, WTW, ACD, and LT measurements were found to be higher in male eyes than female eyes, with a statistically significant difference. The average K1, K2, and AST values were statistically lower in males than females (Table 3).

In the evaluation of the right and left eye ocular parameter averages, statistically significant differences were found in the AL and CCT measurements. Details of the measured parameters are presented in Table 4.

In the correlation analysis of optical biometric measurements, no correlation was found between WTW and CCT, or between K1 and age, AD, ACD, and LT. All other parameters showed either negative or positive correlations with each other (Table 5).

## 4. Discussion

This study constitutes the largest and most representative study conducted on ocular biometric parameters in Türkiye. Although this study covered patients who underwent optical biometry due to cataracts, it can also be considered as providing normal ocular anatomical data for the reasons described below. First, optical biometric devices, including the Lenstar LS900 (Haag-Streit AG, Switzerland) device, are capable of taking measurements in situations with a level of lens clearance that can pass light. However, measurements cannot be taken in patients with advanced cataracts or corneal opacities [4]. Second, some patients have cataracts in one eye but not in the other. Third, in this study, cases where LT or AL could not be measured due to the opacity of the ocular tissues due to advanced cataracts or insufficient light were excluded. Finally, patients who had undergone surgery affecting the anatomy of the eye, including pseudophakic patients, were also excluded from this study. Additionally, the large number of cases belonging to each age group is important from the perspective of anatomical data. The values of ocular optical biometry differ according to age, gender, and ethnicity. Most extensive studies on optical biometrics involve cases with an average age of over 60 (Table 6). In our study, the average age was 56, with cases ranging from three to 110 years of age, and was calculated separately in groups of 20-year increments. Our study, which included detailed data for each age group, addressed a significant gap in the presentation of anatomical normative values of the eye.

The AL measurement was found to be consistent with the literature (Table 6). Two Chinese studies reported higher AL values than other studies [8,9]. Huang et al. found that cataract patients had a significantly higher prevalence of severe axial myopia, defined as AL > 26.5 mm, compared to the normal population, with a rate of 13.66%.

The mean CCT value in the study was 518.13 μm, which is generally lower than the values reported in the literature (Table 6). The CCT value was 517.5 μm [15] in the Japanese population, and 504.2 μm [16] in the healthy Congolese population. These values suggest that CCT may be a genetically determined trait. However, no genes have been identified as contributing to the normal CCT variation seen in the general population [17].

Examining the pattern of changes that occur with age can help us understand the nature of these changes and take appropriate measures when necessary. The eye structure undergoes physiological and pathological changes that can be difficult to distinguish with age. As age progresses, the decrease in the corneal diameter and the resulting narrowing of the anterior chamber may lead to changes in the corneal architecture as a result of compression of the tissues at the angle. The corneal curvature is reported to become steeper with age, which may be due to some physiological changes that change the elasticity of the cornea and cause it to become steeper [18]. A significant decrease in the CCT has also been reported with age. Mashige et al. has suggested that the decrease in keratocyte density with age is responsible for the decrease in CCT values [18]. Some researchers have reported that changes in corneal curvature and power are associated with the process of emmetropization from childhood, and that these changes lead to the steepening of corneal curvature and astigmatism in older adults [19,20]. We observed that all biometric parameters changed with age. As age progresses, AL values decrease (except for the 19 years and under age group), the CCT becomes thinner, the AD narrows, the WTW decreases, the LT thickens, and the K1, K2, and AST decrease toward the age of 60 and then increase again. Jamali et al. reported that nearly all biometric parameters, including the AL, ACD, LT, WTW distance, and AST, are associated with age-related changes. In this context, they found that younger participants exhibited a greater AL, deeper ACD, thinner LT, and larger WTW than older participants. Furthermore, they reported that the average values of most biometric parameters varied between gender subgroups [21]. These findings underscore the importance of considering patient age and gender when interpreting biometric data for ocular diseases and interventions.

In the evaluation of the average ages of males and females, statistically significant differences were identified. However, these minimal differences are not clinically significant enough to impact the results. The biometric measurements of male eyes are statistically larger than female eyes. Accordingly, the keratometry values in female eyes are steeper and the average astigmatism value is higher. However, no significant difference was found between male and female eyes in terms of the average CCT value we determined. In contrast to our study, Nomura et al. reported that the average CCT value was 518.3 µm in males and 511.1 µm in females in their study using a specular microscope [22]. Shimmyo et al. reported that the average CCT value of male corneas (553.98 µm) was significantly thicker than female corneas (547.72 µm) (*p* < 0.001) [23]. However, these differences are not clinically significant. Popov et al. reported differences of 0.44 mm in the AL, 3.11 µm in the CCT, 0.12 mm in the WTW, 0.12 mm in the LT, 0.11 mm in the ACD, 0.62 D in the KM, and 0.01 D in the AST between male eyes and female eyes [14]. These differences are generally consistent with our results. However, in our study, the LT difference was found to be 0.02 mm greater in males than in females. The AL, ACD, and keratometry values are generally used for intraocular lens measurement formulas in cataract surgery. These values have been further examined in optical biometric measurements in the literature, and differences in optical biometric measurements between the genders have been reported in line with our study [5,7,11]. Iyamu and Osuobeni found that among healthy Nigerians, men had significantly larger horizontal corneal diameters than women. They concluded that this finding could be explained by the fact that men generally have longer and, therefore, larger eyes than women [24].

In the evaluation of the right and left eye ocular parameter averages, statistically significant differences were found in the AL and CCT measurements. However, these differences are not clinically significant. In one study, no significant difference was found between the right (551.16 µm) and left eyes (552.55 µm) for the mean CCT [23]. Conversely, Foster et al. found a significant difference (*p* < 0.001) between the right eye (495 µm) and left eye (514 µm) for the mean CCT value [25]. Although there are variations depending on other measurements of the eye, each millimeter difference in AL corresponds to approximately 3 diopters in the intraocular lens measurement [4]. Artificial intraocular lenses are available in different diopters at 0.5 diopter intervals for patients scheduled for cataract surgery. Therefore, AL differences of approximately 0.167 mm and above are considered clinically significant. In this study, the mean AL difference between the right and left eyes was found to be 0.07 mm. In addition to AL measurement, keratometry measurement is also effective in determining the intraocular lens power. In the keratometry values we obtained, no statistically or clinically significant difference was detected between the right and left eyes. Therefore, in eyes with advanced cataracts or in which intraocular lens measurement cannot be performed for other reasons, other eye measurements can be used to give an idea about the use of intraocular lenses.

We found no correlation between the WTW and CCT. Furthermore, the K1 value was not affected by age, but the AD, ACD, and LT, and all other measurements were correlated with each other and age. Naturally, a positive correlation is expected between the AL, WTW and AD. In our study, the AL showed a negative correlation with the LT. In one previous study, a higher LT was found to be associated with a shorter AL [26]. Meng et al. reported that with the extension of the AL, the LT first slightly increased to a maximum of 4.75 ± 0.45 mm in the 20.01–22 mm AL group, then gradually decreased to a minimum of 4.33 ± 0.45 mm in the 26.01–28 mm AL group, and finally increased again and reached a peak when the AL was greater than 35 mm. They noted that AL elongation is associated with myopia and that moderately myopic eyes tend to have thinner lenses than emmetropic eyes [8]. The thinning of the lens in myopic eyes may indicate an attempt to control the overall refractive state of the lens toward emmetropia or may be a result of an attempt to obtain a clear image on the retina [27].

Negative correlations between the CCT and the AL, AD, LT, K1, K2, and AST were observed. Contrary to the findings of Popov et al., which showed no relationship between the AL and CCT, it was observed that the CCT decreased as the AL increased [14]. Similarly, a study show that the CCT decreased in myopic eyes [28]. Another study suggested that a thicker lens is associated with a thicker cornea [8]. However, a negative correlation between the CCT and LT was observed.

The relationship between increasing age and LT is consistent with previous a study supporting an age-related increase in thickness due to the continuous production of new lens fibers in the equatorial region of the lens [26]. In this context, a strong negative correlation between the LT and AD was observed. With the progression of cataracts, the lens tends to thicken bilaterally, which may result in a shallower anterior chamber [26].

In terms of the relationship between CCT and K1 and K2, it was reported that eyes with thicker corneas are flatter and eyes with thinner corneas are steeper [23]. Our study showed that eyeballs with larger AL were associated with flatter corneal curvature, thinner CCT, and profound AD findings. This relationship is consistent with the literature [29]. However, it has also been reported that the CCT is not associated with refractive error, keratometry, AD/ACD, or AL. The CCT has been defined as an independent factor with no relationship to other ocular parameters [29]. These discrepancies can be explained by different methodologies. Although collagen is the main component of the sclera and cornea, differences were observed in glycosaminoglycan and elastin content, hydration, and the predominant collagen type [30]. The CCT may not be affected by the scleral thinning that occurs during globe elongation.

Thanks to optical biometry devices, eye measurements now provide more detailed and accurate data. These devices are continuously evolving, thereby enhancing our understanding of eye diseases. The cross-sectional imaging capabilities of optical imaging techniques enable emerging applications, such as optoretinography, which facilitates the acquisition of both structural and functional information about the eye [31,32].

The most important limitations of our study are that it is retrospective, individuals with and without cataracts cannot be distinguished due to the lack of detailed ophthalmological examination findings, and follow-up data are unavailable due to it being a cross-sectional study. Another limitation of this study is the change in population biometrics over the years; as individuals grow taller, their ocular parameters may also change.

## 5. Conclusions

This study presents ocular biometric parameters with the largest number of cases in Türkiye. No clinically significant difference was found between the right and left eyes. Male eyes were determined to be larger than female eyes in terms of the AL, AD, WTW, and LT, and the cornea was flatter and astigmatism lower. The optical biometric measurements were observed to be generally related to each other and changed with age. These data may be useful in explaining the anatomical relationships of the eye. By clarifying the risk factors of diseases related to ocular measurements, this research may help in planning more appropriate approaches against such diseases.

## Figures and Tables

**Table 1:** Distribution of subjects by age group, gender, and right–left eye.

		Age Group (Year)					Total
		≤19	20–39	40–59	60–79	≥80	
Gender *n* (*%*)	Male	652 (8.2%)	1009 (12.7%)	1943 (24.5%)	3903 (49.2%)	421 (5.3%)	7928 (100.0%)
	Female	428 (6.8%)	873 (14.0%)	1345 (21.5%)	3129 (50.0%)	480 (7.7%)	6255 (100.0%)
Eye *n* (*%*)	OD	494 (7.2%)	931 (13.5%)	1591 (23.2%)	3413 (49.7%)	442 (6.4%)	6871 (100.0%)
	OS	586 (8.0%)	951 (13.0%)	1697 (23.2%)	3619 (49.5%)	459 (6.3%)	7312 (100.0%)
Total		1080 (7.6%)	1882 (13.3%)	3288 (23.2%)	7032 (49.6%)	901 (6.4%)	14,183 (100.0%)

OD: right eye; OS: left eye.

**Table 2:** Optical biometric measurements by age group (the differences between age groups that present different letters are statistically significant; *p* < 0.05).

		Mean ± Std. Dev.	95% Confidence Interval		Min.	Max.	*p*
			Lower	Upper			
AL (mm)	≤19	23.14 ± 1.33 ^d^	23.06	23.22	19.30	31.56	0.001
	20–39	24.03 ± 1.79 ^a^	23.95	24.11	18.63	31.99	
	40–59	23.70 ± 1.66 ^b^	23.64	23.76	18.09	31.97	
	60–79	23.47 ± 1.24 ^c^	23.44	23.50	18.71	33.47	
	≥80	23.45 ± 1.17 ^c^	23.38	23.53	20.43	31.37	
	Total	23.57 ± 1.45	23.55	23.6	18.09	33.47	
CCT (μm)	≤19	527.91 ± 46.94 ^a^	525.1	530.71	346	701	0.001
	20–39	520.74 ± 42.90 ^b^	518.8	522.68	304	735	
	40–59	521.27 ± 34.12 ^b^	520.1	522.44	362	652	
	60–79	515.34 ± 36.09 ^c^	514.5	516.19	347	735	
	≥80	511.24 ± 36.60 ^d^	508.85	513.64	408	695	
	Total	518.13 ± 37.81	517.51	518.75	304	735	
AD (mm)	≤19	3.11 ± 0.36 ^a^	3.09	3.13	1.77	5.46	0.001
	20–39	3.05 ± 0.36 ^b^	3.04	3.07	1.55	5.00	
	40–59	2.79 ± 0.37 ^c^	2.78	2.80	1.55	4.74	
	60–79	2.64 ± 0.39 ^d^	2.63	2.65	1.51	5.28	
	≥80	2.52 ± 0.40 ^e^	2.49	2.55	1.56	4.85	
	Total	2.76 ± 0.42	2.75	2.76	1.51	5.46	
WTW (mm)	≤19	12.19 ± 0.57 ^a^	12.15	12.22	8.05	15.61	0.001
	20–39	12.12 ± 0.48 ^b^	12.10	12.15	9.61	14.04	
	40–59	11.92 ± 0.52 ^c^	11.9	11.94	8.89	15.26	
	60–79	11.78 ± 0.58 ^d^	11.76	11.79	7.12	14.50	
	≥80	11.62 ± 0.78 ^e^	11.56	11.67	7.75	14.78	
	Total	11.88 ± 0.59	11.87	11.89	7.12	15.61	
ACD (mm)	≤19	3.64 ± 0.35 ^a^	3.62	3.66	2.29	5.92	0.001
	20–39	3.57 ± 0.35 ^b^	3.56	3.59	2.12	5.57	
	40–59	3.31 ± 0.37 ^c^	3.3	3.32	2.09	5.23	
	60–79	3.15 ± 0.39 ^d^	3.14	3.16	1.96	5.72	
	≥80	3.03 ± 0.40 ^e^	3.01	3.06	2.08	5.36	
	Total	3.27 ± 0.42	3.27	3.28	1.96	5.92	
LT (mm)	≤19	3.44 ± 0.30 ^e^	3.42	3.46	2.51	5.88	0.001
	20–39	3.70 ± 0.34 ^d^	3.69	3.72	2.57	5.81	
	40–59	4.15 ± 0.40 ^c^	4.13	4.16	2.66	6.38	
	60–79	4.41 ± 0.40 ^b^	4.40	4.42	2.50	6.14	
	≥80	4.55 ± 0.42 ^a^	4.52	4.58	2.71	6.45	
	Total	4.19 ± 0.51	4.18	4.2	2.50	6.45	
K1 (D)	≤19	43.52 ± 2.55 ^a^	43.37	43.67	35.69	65.71	0.001
	20–39	43.31 ± 2.67 ^c^	43.19	43.43	33.39	72.02	
	40–59	43.26 ± 1.81 ^c^	43.20	43.33	31.07	55.60	
	60–79	43.43 ± 1.71 ^b^	43.39	43.47	32.46	59.17	
	≥80	43.47 ± 1.76 ^a^	43.35	43.59	36.72	48.44	
	Total	43.39 ± 1.96	43.35	43.42	31.07	72.02	
K2 (D)	≤19	45.15 ± 3.51 ^a^	44.94	45.36	37.02	77.25	0.001
	20–39	44.75 ± 3.51 ^b^	44.59	44.90	33.77	79.27	
	40–59	44.24 ± 1.97 ^d^	44.17	44.30	32.87	65.18	
	60–79	44.45 ± 1.80 ^c^	44.41	44.5	36.99	66.60	
	≥80	44.68 ± 1.78 ^b^	44.56	44.79	38.08	55.06	
	Total	44.51 ± 2.31	44.47	44.55	32.87	79.27	
AST (D)	≤19	1.63 ± 1.68 ^a^	1.53	1.73	0	12.18	0.001
	20–39	1.44 ± 1.53 ^b^	1.37	1.51	0	12.87	
	40–59	0.97 ± 0.93 ^d^	0.94	1.01	0	10.35	
	60–79	1.02 ± 0.98 ^d^	1.0	1.04	0	14.36	
	≥80	1.20 ± 1.06 ^c^	1.13	1.28	0	8.65	
	Total	1.12 ± 1.15	1.11	1.14	0	14.36	

AL: axial length, CCT: central corneal thickness, AD: humor aqueous depth (between the corneal endothelium and anterior crystalline lens surface), WTW: white-to-white (horizontal corneal diameter), ACD: anterior camera depth (between the corneal epithelium and anterior crystalline lens surface), LT: lens thickness, K1: flat keratometry, K2: steep keratometry, AST: astigmatism, D: diopters.

**Table 3:** Optical biometric measurements by gender (female and male).

		Mean ± Std. Dev.	95% Confidence Interval		Min.	Max.	*p*
			Lower	Upper			
Age (Year)	Male	55.76 ± 20.06	55.31	56.20	3	96	0.001
	Female	57.07 ± 20.17	56.57	57.57	3	110	
AL (mm)	Male	23.71 ± 1.26	23.68	23.73	18.63	33.47	0.001
	Female	23.40 ± 1.64	23.36	23.44	18.09	31.99	
CCT (μm)	Male	517.95 ± 38.02	517.11	518.79	304	735	0.522
	Female	518.36 ± 37.52	517.43	519.29	347	735	
AD (mm)	Male	2.81 ± 0.41	2.80	2.82	1.53	5.46	0.001
	Female	2.69 ± 0.42	2.68	2.70	1.51	5.08	
WTW (mm)	Male	11.94 ± 0.60	11.92	11.95	7.12	15.61	0.001
	Female	11.80 ± 0.56	11.79	11.81	7.62	14.72	
ACD (mm)	Male	3.33 ± 0.41	3.32	3.33	1.96	5.92	0.001
	Female	3.21 ± 0.42	3.20	3.22	2.00	5.62	
LT (mm)	Male	4.20 ± 0.52	4.19	4.21	2.50	6.45	0.001
	Female	4.18 ± 0.49	4.16	4.19	2.51	6.01	
K1 (D)	Male	43.04 ± 1.94	43.00	43.08	31.07	72.02	0.001
	Female	43.82 ± 1.90	43.77	43.87	32.23	61.30	
K2 (D)	Male	44.13 ± 2.29	44.08	44.18	32.87	79.27	0.001
	Female	44.99 ± 2.25	44.93	45.05	33.77	69.92	
AST (D)	Male	1.09 ± 1.13	1.07	1.12	0.00	12.73	0.001
	Female	1.17 ± 1.18	1.14	1.20	0.00	14.36	

AL: axial length, CCT: central corneal thickness, AD: humor aqueous depth (between the corneal endothelium and anterior crystalline lens surface), WTW: white-to-white (horizontal corneal diameter), ACD: anterior camera depth (between the corneal epithelium and anterior crystalline lens surface), LT: lens thickness, K1: flat keratometry, K2: steep keratometry, AST: astigmatism, D: diopters.

**Table 4:** Optical biometric measurements for the right and left eyes.

		Mean ± Std. Dev.	95% Confidence Interval		Min	Max.	*p*
			Lower	Upper			
Age (Year)	OD	56.41 ± 19.91	55.94	56.88	3	110	0.651
	OS	56.26 ± 20.32	55.79	56.73	3	96	
	Total	56.33 ± 20.12	56.00	56.67	3	110	
AL (mm)	OD	23.61 ± 1.46	23.58	23.65	18.63	33.47	0.003
	OS	23.54 ± 1.44	23.50	23.57	18.09	33.12	
	Total	23.57 ± 1.45	23.55	23.58	18.09	33.47	
CCT (μm)	OD	517.42 ± 37.59	516.53	518.31	304	711	0.030
	OS	518.79 ± 37.99	517.92	519.67	346	735	
	Total	518.13 ± 37.81	517.51	518.75	304	735	
AD (mm)	OD	2.76 ± 0.42	2.75	2.7706	1.51	5.28	0.262
	OS	2.75 ± 0.42	2.74	2.7623	1.52	5.46	
	Total	2.76 ± 0.42	2.75	2.7635	1.51	5.46	
WTW (mm)	OD	11.87 ± 0.58	11.86	11.89	7.73	14.78	0.598
	OS	11.88 ± 0.59	11.86	11.89	7.12	15.61	
	Total	11.88 ± 0.59	11.87	11.89	7.12	15.61	
ACD (mm)	OD	3.29 ± 0.42	3.27	3.29	1.99	5.72	0.346
	OS	3.27 ± 0.42	3.26	3.28	1.96	5.92	
	Total	3.27 ± 0.42	3.27	3.28	1.96	5.92	
LT (mm)	OD	4.18 ± 0.51	4.17	4.19	2.51	6.45	0.057
	OS	4.20 ± 0.51	4.19	4.21	2.50	6.36	
	Total	4.19 ± 0.51	4.18	4.20	2.50	6.45	
K1 (D)	OD	43.38 ± 1.96	43.33	43.42	31.07	72.02	0.584
	OS	43.39 ± 1.96	43.35	43.44	32.46	65.71	
	Total	43.39 ± 1.96	43.35	43.42	31.07	72.02	
K2 (D)	OD	44.49 ± 2.32	44.43	44.54	32.87	79.27	0.230
	OS	44.53 ± 2.31	44.48	44.59	33.75	77.25	
	Total	44.51 ± 2.31	44.47	44.55	32.87	79.27	
AST (D)	OD	1.11 ± 1.17	1.08	1.14	0.0	14.36	0.139
	OS	1.14 ± 1.13	1.11	1.16	0.0	12.87	
	Total	1.12 ± 1.15	1.10	1.14	0.0	14.36	

OD: right eye, OS: left eye, AL: axial length, CCT: central corneal thickness, AD: humor aqueous depth (between the corneal endothelium and anterior crystalline lens surface), WTW: white-to-white (horizontal corneal diameter), ACD: anterior camera depth (between the corneal epithelium and anterior crystalline lens surface), LT: lens thickness, K1: flat keratometry, K2: steep keratometry, AST: astigmatism, D: diopters.

**Table 5:** Correlation distribution of optical biometric measurements (**: *p* < 0.01).

Correlations											
		Age	AL	CCT	AD	WTW	ACD	LT	K1	K2	AST
Age	r	1									
	*p*										
AL	r	−0.033 **	1								
	*p*	0.0001									
CCT	r	−0.116 **	−0.036 **	1							
	*p*	0.0001	0.0001								
AD	r	−0.434 **	0.332 **	−0.087 **	1						
	*p*	0.0001	0.0001	0.0001							
WTW	r	−0.275 **	0.214 **	0.010	0.368 **	1					
	*p*	0.0001	0.0001	0.250	0.0001						
ACD	r	−0.446 **	0.330 **	0.002	0.996 **	0.370 **	1				
	*p*	0.0001	0.0001	0.795	0.0001	0.0001					
LT	r	0.654 **	−0.130 **	−0.030 **	−0.609 **	−0.187 **	−0.614 **	1			
	*p*	0.0001	0.0001	0.0001	0.0001	0.0001	0.0001				
K1	r	0.001	−0.321 **	−0.200 **	0.014	−0.272 **	−0.003	0.015	1		
	*p*	0.896	0.0001	0.0001	0.090	0.0001	0.682	0.075			
K2	r	−0.070 **	−0.232 **	−0.251 **	0.077 **	−0.239 **	0.054 **	−0.057 **	0.868 **	1	
	*p*	0.0001	0.0001	0.0001	0.0001	0.0001	0.0001	0.0001	0.0001		
AST	r	−0.142 **	0.082 **	−0.163 **	0.129 **	−0.016	0.115 **	−0.140 **	0.039 **	0.530 **	1
	*p*	0.0001	0.0001	0.0001	0.0001	0.067	0.0001	0.0001	0.0001	0.0001	

AL: axial length, CCT: central corneal thickness, AD: humor aqueous depth (between the corneal endothelium and anterior crystalline lens surface), WTW: white-to-white (horizontal corneal diameter), ACD: anterior camera depth (between the corneal epithelium and anterior crystalline lens surface), LT: lens thickness, K1: flat keratometry, K2: steep keratometry, AST: astigmatism.

**Table 6:** Values of optical biometric measurements of the eye from studies conducted across different geographic regions and ethnic backgrounds.

Study	Country	Measurement Method	Age (Year)	AL (mm)	CCT (μm)	AD (mm)	WTW (mm)	ACD (mm)	LT (mm)	K1 (D)	K2 (D)	K (D)	AST (D)
Our study	Türkiye	Lenstar	56.41 (3–110)	23.57	518.13	2.76	11.88	3.27	4.19	43.39	44.51		1.12
Hernández-López et al., 2021 [5]	Cuba	IOL Master	68.7	23.52	-	-	-	3.02	4.55	43.47	44.61	44.04	1.22
Ferreira et al., 2017 [7]	Portugal	Lenstar	69 (44–99)	23.87	-	-	12.02	3.25	4.32	-	-	43.91	1.08
Meng et al., 2021 [8]	China	IOL Master	62.5 (18–101)	24.71	550	2.52	11.7	-	4.51	-	-	43.89	-
Huang et al., 2018 [9]	China	IOL Master	(50–98)	24.32	-	-	-	3.08	-	-	-	44.23	1.0
Lim et al., 2010 [10]	Singapore	IOL Master	57.3 (40–80)	23.55	-	-	-	3.10	-	-	-	44.12	-
Fotedar et al., 2010 [11]	Australia	IOL Master	(59–85+)	23.44	-	-	12.06	3.24	-	43.38	-	43.42	-
Lee et al., 2009 [12]	USA	IOL Master	71.9 (58–100)	23.69	-	-	-	3.11	-	-	-	43.83	-
Tamaoki et al., 2019 [13]	Japan	IOL Master	71.95	24.09	550	-	-	3.09	4.62	-	-	44.48	-
Popov et al., 2022 [14]	Slovakya	Lenstar	70.35 (19–96)	23.33	551	-	11.96	3.08	4.57	-	-	44.03	0.91

AL: axial length, CCT: central corneal thickness, AD: humor aqueous depth (between the corneal endothelium and anterior crystalline lens surface), WTW: white-to-white (horizontal corneal diameter), ACD: anterior camera depth (between the corneal epithelium and anterior crystalline lens surface), LT: lens thickness, K1: flat keratometry, K2: steep keratometry, K: mean keratometry, AST: astigmatism.

## Data Availability

The raw data supporting the conclusions of this article will be made available by the authors upon request.
